# Effect of Sunlight on the Efficacy of Commercial Antibiotics Used in Agriculture

**DOI:** 10.3389/fmicb.2021.645175

**Published:** 2021-06-01

**Authors:** Sebastian J. Khan, Amanda M. Osborn, Prahathees J. Eswara

**Affiliations:** Department of Cell Biology, Microbiology and Molecular Biology, University of South Florida, Tampa, FL, United States

**Keywords:** antibiotic stewardship, antibiotic resistance, *Liberibacter*, huanglongbing, *Erwinia*, fire blight, streptomycin, oxytetracycline

## Abstract

Antibiotic stewardship is of paramount importance to limit the emergence of antibiotic-resistant bacteria in not only hospital settings, but also in animal husbandry, aquaculture, and agricultural sectors. Currently, large quantities of antibiotics are applied to treat agricultural diseases like citrus greening disease (CGD). The two commonly used antibiotics approved for this purpose are streptomycin and oxytetracycline. Although investigations are ongoing to understand how efficient this process is to control the spread of CGD, to our knowledge, there have been no studies that evaluate the effect of environmental factors such as sunlight on the efficacy of the above-mentioned antibiotics. We conducted a simple disc-diffusion assay to study the efficacy of streptomycin and oxytetracycline after exposure to sunlight for 7- or 14-day periods using *Escherichia coli* and *Bacillus subtilis* as the representative strains of Gram-negative and Gram-positive organisms, respectively. Freshly prepared discs and discs stored in the dark for 7 or 14 days served as our controls. We show that the antibiotic potential of oxytetracycline exposed to sunlight dramatically decreases over the course of 14 days against both *E. coli* and *B. subtilis*. However, the effectiveness of streptomycin was only moderately impacted by sunlight. It is important to note that antibiotics that last longer in the environment may play a deleterious role in the rise and spread of antibiotic-resistant bacteria. Further studies are needed to substantively analyze the safety and efficacy of antibiotics used for broader environmental applications.

## Importance

Although antibiotics have been used for agricultural purposes for decades, due to the rapid rise in antibiotic resistance this usage needs to be revisited. Questions remain on the appropriate mode of application for antibiotics and the actual benefits of using antibiotics for treating the infections caused by plant pathogens, especially for the ones that are intracellular in nature. Here, we show that the two commonly used commercial antibiotics, oxytetracycline and streptomycin, lose their efficacy at different rates in the presence of sunlight. While the former loses its potency within days, the latter remains active for many days. Thus, oxytetracycline may not be active long enough to produce its desired effect, and streptomycin may persist in the environment and as a side effect due to its selective pressure, may force the rise of streptomycin-resistant pathogens.

## Introduction

Antibiotic resistance-related mortalities are expected to exceed the other leading causes of death such as cancer worldwide by 2050 (PLoS Medicine Editors, 2016). Antibiotic stewardship is therefore promoted in all sectors including human health, animal husbandry, and agriculture (Thanner et al., 2016; McEwen and Collignon, 2018; Hernando-Amado et al., 2019). The World Health Organization and the United States Centers for Disease Control and Prevention have recognized antimicrobial resistance as an enormous ongoing threat to public health (Toner et al., 2015; Kadri, 2019). Runoff of antibiotics in hospital waste water (Hocquet et al., 2016) and intentional use in aquaculture (Cabello et al., 2016), animal husbandry (Landers et al., 2012; Martin et al., 2015; Van Boeckel et al., 2019), and crop management (Sundin and Wang, 2018) contribute to the rise and spread of antibiotic resistant bacteria. In this context, alarm was raised recently regarding the spraying of antibiotics in open fields as an infection control strategy to stem the spread of bacterial disease in plants (McKenna, 2019; No authors listed, 2019). Specifically, the strategy approved by the United States Environmental Protection Agency (Collins and Kough, 2017; Donley, 2019; McKenna, 2019) is to use streptomycin and oxytetracycline to control the spread of citrus greening disease (CGD), also known as huanglongbing (yellow dragon disease). CGD is a devastating bacterial disease caused by *Candidatus Liberibacter asiaticus* (CLas) that is transmitted between plants by certain psyllids, which are sap-feeding insects. CLas is a fastidious, Gram-negative, intracellular plant pathogen that belongs to the phylum of α-proteobacteria (Merfa et al., 2019; Achor et al., 2020). Streptomycin and oxytetracycline are also used to treat infections caused by another bacterial plant pathogen, *Erwinia amylovora*, which causes fire blight in apples, pears, and other related species (Acimovic et al., 2015). *E. amylovora* has dual growth modes - an epiphytic mode that is readily accessible to external antibiotics and an endophytic mode that is less accessible to external antibiotics (Acimovic et al., 2015). In addition, tetracycline antibiotics including oxytetracycline are used in animal husbandry (Granados-Chinchilla and Rodriguez, 2017) and aquaculture (Leal et al., 2019b). Apart from the uses described above, data also suggests that antibiotics may find their way into and possibly persist in different animal and plant tissues (Poapolathep et al., 2008; Mayerhofer et al., 2009; Al-Rimawi et al., 2019; Araby et al., 2020), which could be an alternate pathway that can lead to the development of antibiotic-resistant bacteria. Thus, a comprehensive knowledge of the fate of antibiotics used in agriculture is urgently needed to hopefully curb the rise and spread of antibiotic resistance.

Although the application of antibiotics to treat CGD inspired us to pursue this study, the primary objective of this report is to investigate the effect of environmental factors, specifically sunlight, on the stability of streptomycin and oxytetracycline. To this end, we conducted a disc-diffusion assay with Gram-negative *Escherichia coli* and Gram-positive *Bacillus subtilis* and monitored the zones of inhibition of antibiotic-containing discs that were exposed to sunlight for a 7- or 14-day period. Discs that were kept in the dark for an equivalent duration or that were freshly prepared served as our controls. Based on our results, we report that sunlight significantly impairs the efficacy of oxytetracycline, but only moderately impacts streptomycin. While short-lived antibiotics may not be active long enough for their intended purpose, stable antibiotics may apply constant selection pressure and create an environment conducive for the emergence of antibiotic-resistant strains (Shentu et al., 2015). Our data provides a window into the life span of commercial antibiotics in nature that we hope highlights the need for further rigorous safety and efficacy investigations regarding the environmental use of antibiotics.

## Results

### Oxytetracycline Loses Its Antibiotic Potential in the Presence of Sunlight Within the Span of a Few Days

**FIGURE 1:**
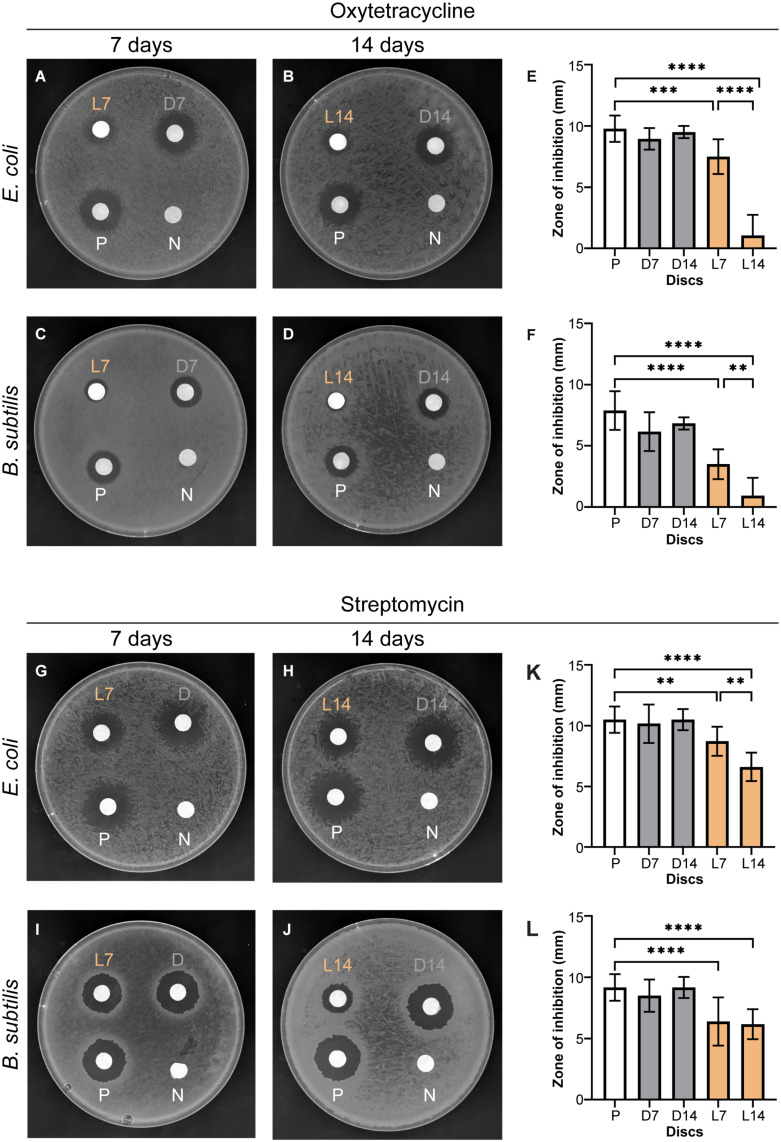
Oxytetracycline and streptomycin lose antibiotic potential in the presence of sunlight. Shown are representative disc-diffusion assay results for the effects of oxytetracycline **(A–D)** or streptomycin **(G–J)** on growth of either Gram-positive *B. subtilis* or Gram-negative *E. coli*. Quantification of the zones of inhibition in millimeters are plotted for each 7- or 14-day cohort of oxytetracycline **(E,F)** and streptomycin **(K,L)**. Significance was determined using a one-way ANOVA with Tukey’s multiple comparisons analysis. Error bars represent standard deviation (SD) of the mean from three biological replicates. N: negative control (discs prepared with sterile water), P: positive control (discs prepared the day of testing), L7 or L14: 7 or 14 days in sunlight, D7 or D14: 7 or 14 days in darkness. ^****^p < 0.0001, ^∗∗∗^*p* < 0.001, ^∗∗^*p* < 0.01.

To monitor the effect of sunlight on the efficacy of oxytetracycline, we conducted a disc-diffusion assay. Briefly, we prepared multiple discs with oxytetracycline (50 μg) dissolved in water and placed the antibiotic-laden discs in either a natural outdoor setting with abundant sunlight to simulate agricultural use, or in a dark indoor cabinet for 7 or 14 days. In addition to the discs that were kept in the dark, we also used freshly prepared discs and vehicle (water) discs as controls. The discs were then placed, as shown in Figure 1, on a pre-inoculated plate containing either a lawn of *E. coli* or *B. subtilis* cells. In all cases, as expected, the blank disc (N; negative control) and the freshly prepared discs (P; positive control) showed negligible and maximum zones of inhibition (ZOI), respectively (Figures 1A–D). The discs that were kept in the dark (labeled “D”) for the duration of 7 or 14 days appeared to produce similar ZOI as our positive control at approximately 9 mm for *E. coli* and 8 mm for *B. subtilis* (Figures 1E,F). This suggests that oxytetracycline maintains its efficiency in the dark at room temperature for at least the maximum duration of this experiment (14 days). Next, we quantified the ZOI for the discs that were exposed to sunlight (labeled “L”) for either a 7- or 14-day period. We observed that the efficacy of oxytetracycline gradually and significantly decreased over time to almost similar to our negative control in both *E. coli* and *B. subtilis* and only retained less than 15% activity after 14 days (Figures 1A–F). This implies that in the presence of sunlight, oxytetracycline loses its antibiotic potential in a matter of a few days.

### Moderate Negative Effects From Sunlight on the Efficacy of Streptomycin

A similar experimental setup to the one discussed above was adopted for studying the effects of sunlight on streptomycin. As noted earlier, blank discs and freshly prepared discs with streptomycin (200 μg) served as our negative and positive controls, respectively. As expected, the ZOI were unobservable for our blank discs and maximum for our positive controls (Figures 1G–L). Similar to oxytetracycline, streptomycin is also able to maintain its efficacy when kept in darkness for the duration of our experiment (Figures 1G–L). However, unlike oxytetracycline, streptomycin appears to be moderately resistant to sunlight. At the 7-day mark, based on the ZOI (Figures 1K,L), the discs exposed to sunlight appear to have retained almost approximately 80 and 70% of their activity in *E. coli* and *B. subtilis*, respectively, when compared to that of our positive control. Further measurable decrease to nearly 50% efficiency compared to our positive control was noted subsequent to 14 days of sunlight exposure for *E. coli*. However, the decrease in efficiency for *B. subtilis* at the 14-day time point was within the standard error when compared to that of the 7-day time point (Figures 1H,J,K,L).

### UV Radiation Is Responsible for the Rapid Decline in the Antibiotic Potential of Oxytetracycline

**FIGURE 2:**
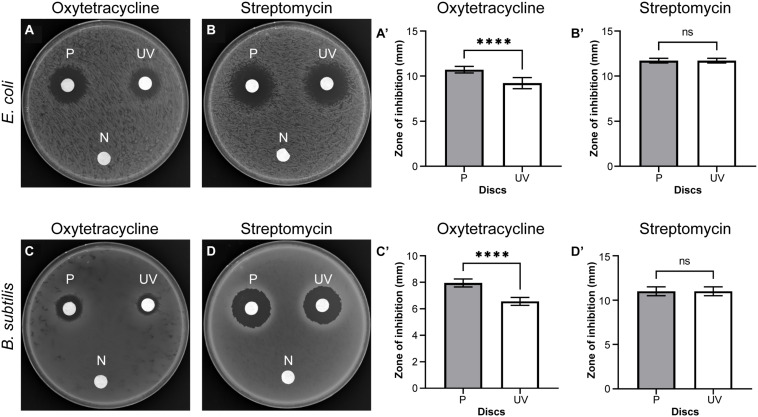
UV irradiation significantly decreases the efficacy of oxytetracycline. Representative results of the disc-diffusion assay for UV-treated oxytetracycline **(A,C)** or streptomycin **(B,D)** conducted using *B. subtilis* and *E. coli* are shown. Quantification of the zones of inhibition in millimeters are plotted for oxytetracycline **(A’,C’)** and streptomycin **(B’,D’).** Significance was determined using a one-way ANOVA with Tukey’s multiple comparisons analysis. Error bars represent standard deviation (SD) of the mean from three biological replicates. N: negative control (discs prepared with sterile water), P: positive control (discs prepared the day of testing), UV: UV-treated discs. ^****^p < 0.0001, ns, not significant.

Next, we investigated whether the increase in temperature or UV radiation from sunlight leads to the increased loss of the antibiotic potential of oxytetracycline. For this purpose, we prepared the antibiotic discs containing oxytetracycline or streptomycin as described previously and placed them either (i) in a dark incubator at 37°C; or (ii) in an enclosed dark chamber illuminated with a UV light source at room temperature; for 48 h. Subsequent to this step, these discs and the control discs were subjected to the disc-diffusion assay described previously. As shown in Figure 2, the blank disc (N; negative control) and the discs that were freshly prepared (P; positive control) showed negligible and maximum ZOI, respectively, in all cases. We noticed that UV-treated oxytetracycline discs displayed a dramatic loss in antibiotic efficacy in both *E. coli* and *B. subtilis* (see Figures 2AA’, 2CC’). Quantification of the ZOI indicated a statistically significant decrease in antibiotic potential upon UV treatment for oxytetracycline. However, this is not the case with streptomycin, as it remains potent even after UV treatment. Heat treatment at 37°C for 48 h also did not significantly alter the antibiotic potential of either antibiotics. For oxytetracycline, the ZOI were 8.00 ± 0.00 mm (P) and 7.95 ± 0.17 mm (heat-treated disc; H) in the case of *B. subtilis* and 10.28 ± 0.26 mm (P) and 10.08 ± 0.48 mm (H) for *E. coli*. For streptomycin, the ZOI were 10.11 ± 0.49 mm (P) and 10.06 ± 0.39 mm (H) for *B. subtilis* and 10.56 ± 0.81 mm (P) and 10.44 ± 0.73 mm (H) for *E. coli*. Based on this data, we infer that UV radiation from sunlight is likely the most dominant cause for the decrease in the antibiotic potential of oxytetracycline.

## Discussion

Rapid rise of antibiotic resistance in bacteria is a major concern worldwide with enormous predicted fatalities resulting from drug-resistant bacteria causing difficult to treat infections. Antibiotics are now routinely used in clinics, animal husbandry, and agriculture. Acknowledgment of the fact that the rise of antibiotic resistance stemming from one of those settings could potentially render antibiotics useless led to the formation of a multidisciplinary collaborative initiative to promote antibiotic stewardship under the umbrella term One Health (McEwen and Collignon, 2018; Hernando-Amado et al., 2019). Despite this, environmental antibiotic pollution is a growing concern that requires urgent attention (Kraemer et al., 2019).

Some commercial antibiotics such as oxytetracycline and streptomycin are produced by soil-dwelling *Streptomyces* spp. However, soil bacteria do not produce antibiotics at levels comparable to commercial applications – which can occasionally be in the scale of thousands of kilograms (Collins and Kough, 2017; Donley, 2019; McKenna, 2019). Also, the efficiency of superficial application of antibiotics in limiting the growth of plant bacterial pathogens, including some that are intracellular, is unclear. Recent studies have suggested injection of oxytetracycline produces better results (Acimovic et al., 2015; Li et al., 2019a). The spread of antibiotic resistance has been documented from agricultural use for antibiotics like tetracycline and streptomycin (Popowska et al., 2012; Tancos et al., 2016; Cycon et al., 2019). It has also been noted that antibiotic resistance genes are naturally found in the environment (Sundin et al., 1995; Schmitt et al., 2006). Therefore, application of consistent selection pressure by excessive and frequent use of antibiotics may enrich the population of naturally resistant organisms. However, at least in some instances under certain conditions, it was noted that streptomycin use did not alter the composition of soil microbial communities appreciably (Shade et al., 2013; Walsh et al., 2013).

Several reports on degradation kinetics and mechanisms of degradation of the antibiotics that are discussed here are available (Wang and Yates, 2008; Xuan et al., 2010; Slana and Dolenc, 2013; Liu et al., 2015; Shen et al., 2017; Leal et al., 2019a,b; Li et al., 2019b; Choi et al., 2020). It has been reported that the half-life of oxytetracycline at 25°C is approximately 7 days, at 35°C is 3 days and at 60°C is 0.2 day, indicating a rapid temperature-dependent degradation of oxytetracycline, as the half-life at 4°C is 120 days (Xuan et al., 2010). According to the same study, the half-life due to photolysis in the presence of sunlight is in the same order of magnitude. A similar investigation exists evaluating the photostability and temperature stability of streptomycin (Shen et al., 2017). Briefly, the photodegradation of streptomycin is more modest than oxytetracycline by nearly 10-fold. The half-life of streptomycin was determined to be nearly 105, 42, and 30 days at 15, 25, and 40°C, respectively, implying a decreased rate of degradation when compared to oxytetracycline. A description of the possible degradation products of oxytetracycline and streptomycin are available (Xuan et al., 2010; Shen et al., 2017). Our results showing a faster loss of efficacy for oxytetracycline than streptomycin upon sunlight exposure are therefore in agreement with the reported degradation kinetics of these antibiotics. To our knowledge, an analysis such as the one we have conducted to monitor the biological efficacy of antibiotics subsequent to exposure to environmental elements are either lacking or not publicly available [as recognized by this article (No authors listed, 2019)]. Our experimental conditions simulate the agricultural use of antibiotics and our results indicate that sunlight contributes to the degradation of oxytetracycline and streptomycin. Our experiments reveal that UV radiation plays a predominant role in the decline of the antibiotic potential of oxytetracycline than heat at conditions tested. Although our report is limited in scope, we believe it sheds light on the fate of antibiotics in the environment. Further studies to understand the effects of antibiotics are needed to inform the public and appropriate regulatory agencies (Thanner et al., 2016; McEwen and Collignon, 2018; Hernando-Amado et al., 2019).

## Materials and Methods

### Strains Used and General Methods

The *B. subtilis* strain PY79 and the *E. coli* strain K-12 were incubated in 2 ml LB at 37°C and grown until the culture OD_600_ reached 1.0 (exponential growth phase). A 100 μl aliquot of culture was then spread onto LB agar plates using sterile beads and set to dry completely prior to the placement of discs, see section below.

### Disc-Diffusion Assay

UV sterilized Whatman filter paper discs (7 mm) were impregnated with 5 μl of a freshly made stock antibiotic solution of either 40 mg/ml streptomycin sulfate (MilliporeSigma) in sterile distilled water or 10 mg/ml oxytetracycline hydrochloride (Alfa Aesar) in sterile distilled water to reach a concentration of 200 μg for streptomycin and 50 μg for oxytetracycline in each disc, and then set to dry completely. The concentrations selected were based on the concentration range recommended for agricultural use (Vidaver, 2002), and after empirically ensuring similar initial zones of inhibition for both antibiotics in the strains tested. To mimic the use of agricultural antibiotics, the discs were then placed outdoors (during the spring months in Tampa, FL, United States where the average daytime temperature ranged from 27 to 32°C) in direct sunlight for 7 or 14 consecutive 24-h periods (days) in parafilm-sealed sterile Petri dishes. Discs that were kept indoors in a dark cabinet at room temperature for 7 or 14 days, freshly prepared discs made the day of testing, and 5 μl of sterile water were used as controls. For the UV experiment, discs were irradiated with a 15 W light source emitting UV radiation at wavelength between 385 and 400 nm at a distance of 9 cm between the light source and the discs for 48 consecutive hours at room temperature. Discs were then transferred and pressed onto the pre-inoculated LB agar plates and incubated overnight at 37°C. The zone of inhibition measurements were taken from the center of the disc to the edge of the zone of inhibition, minus disc radius (3.5 mm).

### Statistical Analysis

GraphPad Prism Software (version 8.3.1) was used to analyze the data. All data represent biological triplicate data with technical replicates. Graphs show mean values and error bars represent standard deviation (SD).

## Data Availability Statement

The raw data supporting the conclusions of this article will be made available by the authors, without undue reservation.

## Author Contributions

SK and PE: conception and design of the study, and writing of the manuscript. SK and AO: data acquisition. SK, AO, and PE: analysis and/or interpretation of the data. All authors contributed to the article and approved the submitted version.

## Conflict of Interest

The authors declare that the research was conducted in the absence of any commercial or financial relationships that could be construed as a potential conflict of interest.

## References

[B1] Achor D. Welker S. Ben-Mahmoud S. Wang C. Folimonova S. Y. Dutt M. (2020). Dynamics of candidatus liberibacter asiaticus movement and sieve-pore plugging in citrus sink cells. *Plant Physiol.* 182 882–891. 10.1104/pp.19.01391 31818905 PMC6997701

[B2] Acimovic S. G. Zeng Q. McGhee G. C. Sundin G. W. Wise J. C. (2015). Control of fire blight (Erwinia amylovora) on apple trees with trunk-injected plant resistance inducers and antibiotics and assessment of induction of pathogenesis-related protein genes. *Front. Plant Sci.* 6:16. 10.3389/fpls.2015.00016 25717330 PMC4323746

[B3] Al-Rimawi F. Hijaz F. Nehela Y. Batuman O. Killiny N. (2019). Uptake, translocation, and stability of oxytetracycline and streptomycin in citrus plants. *Antibiotics (Basel).* 8:196. 10.3390/antibiotics8040196 31717884 PMC6963747

[B4] Araby E. Nada H. G. Abou El-Nour S. A. Hammad A. (2020). Detection of tetracycline and streptomycin in beef tissues using Charm II, isolation of relevant resistant bacteria and control their resistance by gamma radiation. *BMC Microbiol.* 20:186. 10.1186/s12866-020-01868-1867 PMC7325294 32600267

[B5] Cabello F. C. Godfrey H. P. Buschmann A. H. Dolz H. J. (2016). Aquaculture as yet another environmental gateway to the development and globalisation of antimicrobial resistance. *Lancet Infect Dis.* 16 e127–e133. 10.1016/S1473-3099(16)00100-106 27083976

[B6] Choi S. Sim W. Jang D. Yoon Y. Ryu J. Oh J. (2020). Antibiotics in coastal aquaculture waters: occurrence and elimination efficiency in oxidative water treatment processes. *J. Hazard Mater.* 396:122585. 10.1016/j.jhazmat.2020.122585 32298861

[B7] Collins S. Kough J. L. (2017). *Review of GeoLogic/Agrosource’s Analysis of Oxytetracycline’s Safety with Regard to Its Microbiological Effect on Bacteria of Human Health Concern (FDA/CVM Guidance to Industry #152) for Registration on Citrus Crop Group 10–10 [Memorandum].* Washington, D.C: US Environmental Protection Agency.

[B8] Cycon M. Mrozik A. Piotrowska-Seget Z. (2019). Antibiotics in the soil environment-degradation and their impact on microbial activity and diversity. *Front. Microbiol.* 10:338. 10.3389/fmicb.2019.00338 30906284 PMC6418018

[B9] Donley N. (2019). The USA lags behind other agricultural nations in banning harmful pesticides. *Environ. Health* 18:44. 10.1186/s12940-019-0488-480 PMC6555703 31170989

[B10] Granados-Chinchilla F. Rodriguez C. (2017). Tetracyclines in food and feedingstuffs: from regulation to analytical methods, bacterial resistance, and environmental and health implications. *J. Anal. Methods Chem.* 2017:1315497. 10.1155/2017/1315497 28168081 PMC5266830

[B11] Hernando-Amado S. Coque T. M. Baquero F. Martinez J. L. (2019). Defining and combating antibiotic resistance from one health and global health perspectives. *Nat. Microbiol.* 4 1432–1442. 10.1038/s41564-019-0503-509 31439928

[B12] Hocquet D. Muller A. Bertrand X. (2016). What happens in hospitals does not stay in hospitals: antibiotic-resistant bacteria in hospital wastewater systems. *J. Hosp. Infect.* 93 395–402. 10.1016/j.jhin.2016.01.010 26944903

[B13] Kadri S. S. (2019). Key takeaways from the U.S. CDC’s antibiotic resistance threats report for frontline providers. *Crit. Care Med.* 48 939–945. 10.1097/CCM.0000000000004371 32282351 PMC7176261

[B14] Khan S. Osborn A. Eswara P. J. (2020). Effect of sunlight on the efficacy of commercial antibiotics used in agriculture. *bioRxiv [preprint]* 10.1101/2020.07.10.197848 PMC8203823 34140934

[B15] Kraemer S. A. Ramachandran A. Perron G. G. (2019). Antibiotic pollution in the environment: from microbial ecology to public policy. *Microorganisms* 7:180. 10.3390/microorganisms7060180 31234491 PMC6616856

[B16] Landers T. F. Cohen B. Wittum T. E. Larson E. L. (2012). A review of antibiotic use in food animals: perspective, policy, and potential. *Public Health Rep.* 127 4–22. 10.1177/003335491212700103 22298919 PMC3234384

[B17] Leal J. F. Esteves V. I. Santos E. B. H. (2019a). Solar photodegradation of oxytetracycline in brackish aquaculture water: new insights about effects of Ca2+ and Mg2+. *J. Photochem. Photobiol. Chem.* 372 218–225.

[B18] Leal J. F. Santos E. B. H. Esteves V. I. (2019b). Oxytetracycline in intensive aquaculture: water quality during and after its administration, environmental fate, toxicity and bacterial resistance. *Rev. Aquacul.* 11 1176–1194. 10.1111/raq.12286

[B19] Li J. Pang Z. Duan S. Lee D. Kolbasov V. G. Wang N. (2019a). The in planta effective concentration of oxytetracycline against ‘Candidatus Liberibacter asiaticus’ for suppression of citrus huanglongbing. *Phytopathology* 109 2046–2054. 10.1094/PHYTO-06-19-0198-R 31369360

[B20] Li Z.-J. Qi W.-N. Feng Y. Liu Y.-W. Ebrahim S. Long J. (2019b). Degradation mechanisms of oxytetracycline in the environment. *J. Int. Agricul.* 18 1953–1960.

[B21] Liu Y. Bao Y. Cai Z. Zhang Z. Cao P. Li X. (2015). The effect of aging on sequestration and bioaccessibility of oxytetracycline in soils. *Environ. Sci. Pollut. Res. Int.* 22 10425–10433. 10.1007/s11356-015-4190-4197 25721525

[B22] Martin M. J. Thottathil S. E. Newman T. B. (2015). Antibiotics overuse in animal agriculture: a call to action for health care providers. *Am. J. Public Health* 105 2409–2410. 10.2105/AJPH.2015.302870 26469675 PMC4638249

[B23] Mayerhofer G. Schwaiger-Nemirova I. Kuhn T. Girsch L. Allerberger F. (2009). Detecting streptomycin in apples from orchards treated for fire blight. *J. Antimicrob Chemother.* 63 1076–1077. 10.1093/jac/dkp055 19240075

[B24] McEwen S. A. Collignon P. J. (2018). Antimicrobial resistance: a one health perspective. *Microbiol. Spectr.* 6 521–547. 10.1128/microbiolspec.ARBA-0009-2017 29600770 PMC11633550

[B25] McKenna M. (2019). Antibiotics set to flood Florida’s troubled orange orchards. *Nature* 567 302–303. 10.1038/d41586-019-00878-874 30890811

[B26] Merfa M. V. Perez-Lopez E. Naranjo E. Jain M. Gabriel D. W. De La Fuente L. (2019). Progress and obstacles in culturing ‘Candidatus Liberibacter asiaticus’, the bacterium associated with huanglongbing. *Phytopathology* 109 1092–1101. 10.1094/PHYTO-02-19-0051-RVW 30998129

[B27] No authors listed (2019). Spraying diseased citrus orchards with antibiotics could backfire. *Nature* 567:283. 10.1038/d41586-019-00875-7 30890810

[B28] PLoS Medicine Editors (2016). Antimicrobial resistance: is the world unprepared? *PLoS Med.* 13:e1002130. 10.1371/journal.pmed.1002130 27618631 PMC5019402

[B29] Poapolathep A. Poapolathep S. Jermnak U. Imsilp K. Wannapat N. Sugita-Konishi Y. (2008). Muscle tissue kinetics of oxytetracycline following intramuscular and oral administration at two dosages to giant freshwater shrimp (*Macrobrachium rosenbergii*). *J. Vet. Pharmacol. Ther.* 31 517–522. 10.1111/j.1365-2885.2008.00988.x 19000273

[B30] Popowska M. Rzeczycka M. Miernik A. Krawczyk-Balska A. Walsh F. Duffy B. (2012). Influence of soil use on prevalence of tetracycline, streptomycin, and erythromycin resistance and associated resistance genes. *Antimicrob Agents Chemother.* 56 1434–1443. 10.1128/AAC.05766-5711 22203596 PMC3294877

[B31] Schmitt H. Stoob K. Hamscher G. Smit E. Seinen W. (2006). Tetracyclines and tetracycline resistance in agricultural soils: microcosm and field studies. *Microb. Ecol.* 51 267–276. 10.1007/s00248-006-9035-y 16598633

[B32] Shade A. Klimowicz A. K. Spear R. N. Linske M. Donato J. J. Hogan C. S. (2013). Streptomycin application has no detectable effect on bacterial community structure in apple orchard soil. *Appl. Environ. Microbiol.* 79 6617–6625. 10.1128/AEM.02017-2013 23974143 PMC3811482

[B33] Shen Y. Zhao W. Zhang C. Shan Y. Shi J. (2017). Degradation of streptomycin in aquatic environment: kinetics, pathway, and antibacterial activity analysis. *Environ. Sci. Pollut. Res. Int.* 24 14337–14345. 10.1007/s11356-017-8978-8975 28429270

[B34] Shentu J. L. Zhang K. Shen D. S. Wang M. Z. Feng H. J. (2015). Effect from low-level exposure of oxytetracycline on abundance of tetracycline resistance genes in arable soils. *Environ. Sci. Pollut. Res. Int.* 22 13102–13110. 10.1007/s11356-015-4099-4091 25925140

[B35] Slana M. Dolenc M. S. (2013). Environmental risk assessment of antimicrobials applied in veterinary medicine-A field study and laboratory approach. *Environ. Toxicol. Pharmacol.* 35 131–141. 10.1016/j.etap.2012.11.017 23274419

[B36] Sundin G. W. Monks D. E. Bender C. L. (1995). Distribution of the streptomycin-resistance transposon Tn5393 among phylloplane and soil bacteria from managed agricultural habitats. *Can. J. Microbiol.* 41 792–799. 10.1139/m95-109 7585356

[B37] Sundin G. W. Wang N. (2018). Antibiotic resistance in plant-pathogenic bacteria. *Annu. Rev. Phytopathol.* 56 161–180. 10.1146/annurev-phyto-080417-45946 29856934

[B38] Tancos K. A. Villani S. Kuehne S. Borejsza-Wysocka E. Breth D. Carol J. (2016). Prevalence of streptomycin-resistant erwinia amylovora in New York apple orchards. *Plant Dis.* 100 802–809. 10.1094/PDIS-09-15-0960-RE 30688602

[B39] Thanner S. Drissner D. Walsh F. (2016). Antimicrobial resistance in agriculture. *mBio* 7:e002227-15. 10.1128/mBio.02227-2215 PMC4850276 27094336

[B40] Toner E. Adalja A. Gronvall G. K. Cicero A. Inglesby T. V. (2015). Antimicrobial resistance is a global health emergency. *Health Secur.* 13 153–155. 10.1089/hs.2014.0088 26042858 PMC4486712

[B41] Van Boeckel T. P. Pires J. Silvester R. Zhao C. Song J. Criscuolo N. G. (2019). Global trends in antimicrobial resistance in animals in low- and middle-income countries. *Science* 365:eaaw1944. 10.1126/science.aaw1944 31604207

[B42] Vidaver A. K. (2002). Uses of antimicrobials in plant agriculture. *Clin. Infect. Dis.* 34(Suppl. 3), S107–S110. 10.1086/340247 11988880

[B43] Walsh F. Smith D. P. Owens S. M. Duffy B. Frey J. E. (2013). Restricted streptomycin use in apple orchards did not adversely alter the soil bacteria communities. *Front. Microbiol.* 4:383. 10.3389/fmicb.2013.00383 24550889 PMC3908321

[B44] Wang Q. Yates S. R. (2008). Laboratory study of oxytetracycline degradation kinetics in animal manure and soil. *J. Agric. Food Chem.* 56 1683–1688. 10.1021/jf072927p 18257526

[B45] Xuan R. Arisi L. Wang Q. Yates S. R. Biswas K. C. (2010). Hydrolysis and photolysis of oxytetracycline in aqueous solution. *J. Environ. Sci. Health B.* 45 73–81. 10.1080/03601230903404556 20390934

